# Fair allocation of scarce medical resources during COVID-19 pandemic: ethical considerations

**DOI:** 10.31744/einstein_journal/2020AE5775

**Published:** 2020-04-24

**Authors:** Erika Satomi, Polianna Mara Rodrigues de Souza, Beatriz da Costa Thomé, Claudio Reingenheim, Eduardo Werebe, Eduardo Juan Troster, Farah Christina de La Cruz Scarin, Hélio Arthur Bacha, Henrique Grunspun, Leonardo José Rolim Ferraz, Marco Aurelio Scarpinella Bueno, Mario Thadeu Leme de Barros, Pedro Custódio de Mello Borges

**Affiliations:** 1 Hospital Israelita Albert Einstein São PauloSP Brazil Hospital Israelita Albert Einstein , São Paulo , SP , Brazil . *; 2 Universidade Federal de São Paulo São PauloSP Brazil Universidade Federal de São Paulo , São Paulo , SP , Brazil .; 3 Faculdade Israelita de Ciências da Saúde Albert Einstein Hospital Israelita Albert Einstein São PauloSP Brazil Faculdade Israelita de Ciências da Saúde Albert Einstein , Hospital Israelita Albert Einstein , São Paulo , SP , Brazil .

## INTRODUCTION

Many are the uncertainties generated by the potential healthcare resource scarcity in the face of a relevant increase in the number of cases of coronavirus disease 2019 (COVID-19), an infectious disease caused by the novel coronavirus (SARS-CoV-2) of the *Coronaviridae* family. The virus emerged in China, in December 2019, spreading rapidly to other regions of the world, including Brazil. Since March 11, 2020, COVID-19 has been considered a pandemic by the World Health Organization (WHO).

Viral pandemics tend to be a serious threat to the stability of health systems, imposing extraordinary and sustained demands on them, which can exceed the service capacity regarding all their available supplies and technologies, as well as trained human/professional resources. Pandemics pose the enormous challenge of balancing equality of all people and equity in distribution of risks and benefits among them. ^( 1 )^

In view of this scenario of increasingly frequent cases of COVID-19 in the population, there is an urgent need to evaluate best practices for optimizing the use of available means and resources. Thus, considering the imminent risk of unavailability of intensive care beds and respirators for all individuals, affected or not by the disease, it is essential to establish clinical, technical and ethical criteria for the best use of them, to enhance results and generate the best possible benefits. ^( 2 , 3 )^

### How can medical resources be allocated more fairly and ethically during the COVID-19 pandemic?

Some international associations argue that, since the pandemic is an exceptional situation, it must be managed as a crisis situation, *i.e* ., it requires “calamity or disaster medicine” measures. In this case, some ethical precepts must be considered, in order to best apply distributive justice in the appropriate allocation of resources. ^( 4 - 6 )^ Therefore, solid technical and scientific criteria, strict ethical principles and legal considerations must be considered. Fair allocation requires an ethical framework, despite the multiple values involved, which can be adapted and revised, depending on the resources and context at issue.

The lack of advance planning in these situations of resource scarcity risk can lead to waste of resources, inadvertent loss of life, and loss of trust from users and professionals. Healthcare systems and providers must be prepared to make the most of limited resources and reduce damage to people, the health system and society. ^( 7 )^ In addition, the heavy task of making decisions about the allocation of available resources should never be imposed upon the professionals who are in the so-called “front line”, who are already overburdened by the scenario, because of the risk of promoting failures and intensifying work stress and illnesses among these professionals. The health of healthcare personnel must also be protected in this process, since they are essential to tackling this crisis.

Unfortunately, in emergency situations, ethical values that guide individual relationships, such as unrestricted respect for autonomy, care centered on individual values, preferences and needs, and ethical values based on the needs of specific groups, should be carefully weighed, allowing health promotion for the majority of the population, with wise use of scarce resources to minimize morbidity and mortality. ^( 8 , 9 )^

It should be noted, however, that the primary ethical principle to be considered is respect for human dignity of all individuals. Therefore, everyone should have the right to a medical triage, with fair and transparent objective criteria, in addition to access to appropriate information about their health status, the conditions of the care system and the established criteria. They must also receive all necessary support for their condition within the group they were triaged for. It is noteworthy that the Brazilian State, in addition to having as a foundation the aforementioned principle of human dignity (Art. 1, item III Federal Constitution of 1988), has as a legal obligation, *i.e* ., a goal to be achieved, in this case materialized in the allocation of resources, the principle of solidarity (Art. 3, item I) and non-discrimination of birth, race, sex, color, age or any other form of discrimination (Art. 3, item IV) (https://www2.senado.leg.br/bdsf/bitstream/handle/id/518231/CF88_Livro_EC91_2016.pdf). This triage document is based on these goals.

The other fundamental values to be considered when preparing the protocols are:

1. Justice in the distribution of resources, observing:
  - The obligation of care, *i.e* ., to provide adequate care, considering the group the patient was assigned to, and to relieve suffering in any situation.
  - The obligation to manage resources and to balance equality and equity in the distribution of resources, using protocols with well-defined criteria and supported by the other values expressed in this document.
2. Maximization of global benefits in the allocation and use of resources, considering:
  - Prioritizing the maximum benefit for all patients and the largest possible number of people, recognizing that not all patients will benefit with or need the use of all resources; therefore, each resource must be directed to those who can really benefit according to the available clinical evidence.
  - Identifying and prioritizing those who can recover using the scarce resources, over those who are likely to recover without the need to use these specific resources, and those whose recovery is unlikely, even if they undergo intensive care treatment.
  - The factors to be weighed in this decision, which include:
    i. The evaluation of short and long-term life expectancy (considering current illness and previous comorbidities) using validated instruments;
    ii. Estimated life years saved, prioritizing patients who are likely to survive longer after treatment;
    iii. The “right” to live a full life cycle;
    iv. Cases identified as “irreversible”, which should be allocated to receive adequate Palliative Care as soon as possible. ^( 10 )^
3. Consideration of instrumental value:
  - Health professionals are essential to tackle the crisis generated by the pandemic, hence, considering the factors described above, their care should be prioritized, aiming at a faster recovery and return to work capacity. Illness of the workforce, *i.e* ., of health professionals on the front line, is to be taken into consideration. The concept is to save the life of those who can save more lives, because without trained professionals, all patients - not just those with COVID-19 - will have greater risk of mortality and years of life lost.
4. No priority for disease: during a pandemic, patients suffering from other diseases must be triaged by the same criteria for priority of care.
5. Offering adequate Palliative Care to all those who do not meet the criteria for admission to intensive care units (ICU) due to a severe condition, with a low possibility of responding to available treatments and a poor prognosis.

The purpose of this document is to provide a triage guide considering the possibility of the resource demand ( *e.g* ., ventilators, ICU beds) exceeding their availability. It is suggested these recommendations be implemented immediately, with the approval of the Administration/Board of each hospital, and each sector will decide on when and how to implement it, respecting local particularities. Likewise, it is suggested, as long as the exceptional situation imposed by the pandemic lasts, this policy of allocation of resources be maintained, and the decision to discontinue it should be taken together with the Administration/Board of each hospital.

### Decision process for resource allocation

#### Triage

The triage should not be based on a suspected COVID-19 diagnosis, but for everyone in critical condition, providing equal access for all.

The attending physician should not be the one who makes decisions based on the triage criteria. It is recommended that the priority level decision-making process be carried out by at least two members of the hospital administration staff ( *e.g* ., the ICU director or the head of the emergency department). Once the priority level has been defined, the patient or his legal representative must be informed about the resources assigned to the patient. It is also essential that this decision-making process be recorded in the patient’s medical record.

#### Assessment criteria

It is important to note that the triage screening process is not based on ethnicity, purchasing power, perceptions of quality of life, intellectual disability, social status, presence of specific comorbidities or sex. The initial triage is based on survival criteria, using scores, such as the Clinical Frailty Scale, by Rockwood et al., ^( 11 )^ (Table 1). This is a tool to assess physical, cognitive and functional frailty, reflecting both comorbidities and physiological reserve, which can be applied to all adult individuals, regardless of age or type of underlying disease, allowing equity in the assessment. Frailty in ICU patients is a risk factor for in-hospital mortality and also for no home return. ^( 12 , 13 )^ The allocation of resources, then, is based on the score of the selected scale, which defines the degree of priority (Table 2).

**Table 1:** Clinical Frailty Scale by Rockwood et al. ( ^11^ )

Frailty scale	Description
1. Very fit	Robust, active, energetic and motivated. Commonly exercise regularly and are among the fittest for their age
2. Well	Have no active disease, but less fit than category 1
3. Managing well with treated comorbidities	Symptoms related to the underlying disease are well controlled
4. Apparently vulnerable	Although not dependent, usually complaint of being “slowed up” or have symptoms related to the comorbidity
5. Mildly frail	Have some dependence on other people for instrumental activities (transportation, shopping, preparing meals, dealing with finances, using the phone, managing medications and housework)
6. Moderately frail	Need help with basic activities of daily living (feeding, going to the bathroom, choosing clothes, personal hygiene, sphincter continence, dressing, bathing, walking and transferring)
7. Severely frail	Completely dependent on others for basic activities of daily living

**Table 2:** Resource allocation according to the Clinical Frailty Scale, by Rockwood et al. ( ^11^ )

Degree of priority	Rockwood	Allocation
High priority	1-3	ICU/stepdown unit
Intermediate priority	4-6	ICU trial
Low priority	7	Hospital ward – Palliative care

ICU: intensive care unit; ICU trial: attempted treatment in the intensive care unit.

If there is a tie in the score, the decision may be based on the estimated life years saved, and on the criterion of being a health professional involved in the care of patients with COVID-19. The latter does not mean that the life of this professional is more valuable than the life of other people, but derives from its instrumental value, as part of the workforce to assist in the care of other individuals. Participation in clinical studies related to COVID-19 can also be used as a criterion for resource allocation, as a reward for the willingness to be part of a study that can benefit others.

## ICU TRIAL

In cases in which a limited ICU treatment attempt is chosen (ICU trial), it is recommended that the expected improvement is clearly explained upon ICU admission, emphasizing that if this does not happen, the patient will be removed from the ICU due to therapeutic failure. The Sequential Organ Failure Assessment (SOFA) appears to be one of the prognostic factors for in-hospital mortality during the pandemic (Table 3). ^( 14 , 15 )^ Patients with ascending SOFA and, especially, with SOFA >10 should be candidate for therapeutic limitation ( *e.g* ., limitation of vasoactive drug (VAD) infusion, hemodialysis, mechanical ventilation and cardiopulmonary resuscitation maneuvers). According to the patient’s response during mechanical ventilation, at least 7 days of therapy are proposed until evaluating respiratory support withdrawal, which must be done by two healthcare professionals who are not directly involved in the patient’s care, to increase objectivity and reduce moral stress among the staff. In these situations, it is recommended that the Psychology and Palliative Care teams are called in, in addition to allowing family members to attend the patient’s final phase of life (limited to two accompanying persons) in person or by videoconference.

**Table 3:** Sequential Organ Failure Assessment Score

SOFA	0	1	2	3	4
PaO _2_ /FiO _2_ , mmHg	≥400	<400	<300	<200 (with ventilatory support)	<100 (with ventilatory support)
Platelets, x10 ^3^ /mm ^3^	≥150	<150	<100	<50	<20
Bilirubin, mg/dL	<1.2	1.2-1.9	2.0-5.9	6.0-11.9	≥12.0
Cardiovascular drugs in µg/kg/minute	MAP ≥70mmHg	MAP <70mmHg	Dopamine ≤5 or dobutamine (any dose)	Dopamine 5-15 or epinephrine ≤0.1 or norepinephrine ≤0.1	Dopamine >15 or epinephrine >0.1 or norepinephrine >0.1
Glasgow coma scale	15	13-14	10-12	6-9	≤6
Creatinine, mg/dL Urinary output	<1.2	1.2-1.9	2.0-3.4	3.5-4.9 or diuresis <500mL/d	≥5.0 or diuresis <200mL/d

SOFA: Sequential Organ Failure Assessment; PaO _2_ /FiO _2_ : partial pressure of oxygen/fraction of inspired oxygen; MAP: mean arterial pressure.

## Low priority individuals

Those allocated as low priority should receive symptom control measures and psychosocial support in a hospital ward. The Palliative Care team can assist in the coordination of symptom control measures, with the attending physician, and facilitate the communication process with the patient and his family.

In situations of imminent death, the attendance of two accompanying persons or a videoconference is suggested.

In situations in which the patient, or his legal representative, requests to leave the hospital, to avoid social isolation, even with a high risk of mortality, and to be in the company of his family members, we recommend that the Palliative Care team is called in. The team has the role of advising the patient and the family on the risks and benefits of this option, in addition to clarifying possible strategies for symptom control and post-death care.
