# Supplementary material for: Fair allocation of scarce medical resources during COVID-19 pandemic: ethical considerations
Source: Einstein (Sao Paulo). 2020 Apr 24;18:eAE5775. doi: 10.31744/einstein_journal/2020AE5775 (PMC7186000; doi:10.31744/einstein_journal/2020AE5775)
Supplement: Translation

# Translation: Alocação justa de recursos de saúde escassos diante da pandemia de COVID-19: considerações éticas

**Authors:** Erika Satomi, Polianna Mara Rodrigues de Souza, Beatriz da Costa Thomé, Claudio Reingenheim, Eduardo Werebe, Eduardo Juan Troster, Farah Christina de La Cruz Scarin, Hélio Arthur Bacha, Henrique Grunspun, Leonardo José Rolim Ferraz, Marco Aurelio Scarpinella Bueno, Mario Thadeu Leme de Barros, Pedro Custódio de Mello Borges

**Affiliations:** 1 São PauloSP Brasil Hospital Israelita Albert Einstein, São Paulo , SP , Brasil . *; 2 São PauloSP Brasil Universidade Federal de São Paulo, São Paulo , SP , Brasil .; 3 São PauloSP Brasil Faculdade Israelita de Ciências da Saúde Albert Einstein, Hospital Israelita Albert Einstein, São Paulo , SP , Brasil .

## INTRODUÇÃO

Muitas são as incertezas geradas pela possibilidade de escassez de recursos em saúde ante o aumento relevante de casos de *coronavirus disease* 2019 (COVID-19), gerada por infeção pelo novo coronavírus tipo 2 (SARS-CoV-2) da família *Coronaviridae* , que emergiu na China, em dezembro de 2019, espalhando-se rapidamente para outras regiões do mundo, incluindo o Brasil. Desde 11 de março de 2020, a COVID-19 passou a ser considerada uma pandemia pela Organização Mundial de Saúde (OMS).

As pandemias virais costumam representar sérias ameaças ao equilíbrio dos sistemas de saúde, ao impor-lhes demandas extraordinárias e sustentadas, que podem ultrapassar a capacidade de atendimento, no que diz respeito tanto aos insumos e às tecnologias disponíveis, quanto aos recursos humanos/profissionais capacitados. Trazem o enorme desafio de equilibrar igualdade entre as pessoas e equidade na distribuição de riscos e benefícios entre elas.^( 1 )^

Diante desse cenário de casos cada vez mais frequentes de COVID-19 na população, surge a premente necessidade de se avaliarem as melhores práticas para a otimização da utilização dos meios e recursos disponíveis. Dessa forma, considerando o iminente risco de indisponibilidade de leitos de terapia intensiva e respiradores para todos os indivíduos, acometidos ou não pela doença, torna-se fundamental estabelecer critérios clínicos, técnicos e éticos, para a melhor utilização dos mesmos, para se conseguir otimizar resultado e gerar o maior benefício possível.^( 2 , 3 )^

### Como os recursos médicos podem ser alocados de maneira mais justa e ética durante a pandemia de COVID-19?

Algumas associações internacionais advogam que, constituindo a pandemia uma situação excepcional, ela deve ser gerenciada tal qual uma situação de crise, ou seja, exige medidas de “medicina de catástrofes ou calamidades”. Nesse caso, alguns preceitos éticos devem ser considerados, para que se aplique da melhor forma a justiça distributiva na alocação adequada de recursos.^( 4 - 6 )^Para tanto, devem ser considerados critérios técnicos e científicos sólidos, princípios éticos rigorosos e considerações legais. A alocação justa requer uma estrutura ética, apesar dos múltiplos valores, que podem ser adaptados e revisados, dependendo dos recursos e do contexto em questão.

A falta de planejamento antecipado nessas situações de risco de escassez de recursos pode levar a desperdício de recursos, perda inadvertida de vidas e perda da confiança de usuários e profissionais. Os sistemas e os prestadores de cuidados de saúde devem estar preparados para aproveitarem ao máximo os recursos limitados e reduzirem os danos às pessoas, ao sistema de saúde e à sociedade.^( 7 )^Além disso, o peso das decisões sobre a alocação dos recursos disponíveis não deve recair sobre os profissionais que estão na chamada “linha de frente”, já sobrecarregados pelo cenário que se impõem, sob o risco de serem promovidas falhas e de o estresse e o adoecimento profissional serem intensificados. A integridade dos profissionais de saúde também deve ser protegida nesse processo, uma vez que eles são fundamentais ao enfrentamento da questão.

Infelizmente, em situações emergenciais, impõe-se a necessidade de se pesarem valores éticos que norteiam relações individuais, como respeito irrestrito à autonomia, cuidados centrados em valores, preferências e necessidades individuais, e valores éticos de necessidades de grupos, que permitam promover a saúde da maior parte da população, por meio do uso prudente de recursos escassos, de forma a minimizar a morbidade e a mortalidade.^( 8 , 9 )^

Deve-se destacar, no entanto, que o princípio ético primordial a ser considerado continua sendo o respeito à dignidade humana de todos os indivíduos. Para tanto, todos devem ter o direito à triagem, com critérios objetivos justos e transparentes, além de acesso a informações adequadas sobre seu estado de saúde, as condições do sistema de atendimento e dos próprios critérios estabelecidos. Devem também receber todo suporte necessário para seu quadro dentro do grupo para o qual foi triado. Ressalta-se que o Estado Brasileiro, além de ter como fundamento o já citado princípio da dignidade da pessoa humana (Art. 1º, III Constituição Federal de 1988), tem como dever jurídico, isto é, meta a ser alcançada, no que se materializa na alocação de recursos, o princípio da solidariedade (Art. 3º, I) e da não discriminação de origem, raça, sexo, cor, idade e qualquer outra forma de discriminação (Art. 3º, IV) (https://www2.senado.leg.br/bdsf/bitstream/handle/id/518231/CF88_Livro_EC91_2016.pdf). Este documento de triagem se pauta nessas metas.

Os demais valores fundamentais a serem considerados na construção dos protocolos são:

1. Justiça na distribuição dos recursos, observando:
  - O dever do cuidado, isto é, de prestar cuidados adequados, considerando o grupo para o qual o paciente foi designado e aliviar sofrimento em qualquer situação.
  - O dever de gerenciar os recursos e de equilibrar igualdade e equidade na distribuição dos recursos, por meio de protocolos com critérios bem definidos e apoiados nos demais valores expressos neste documento.
2. Maximização dos benefícios globais na alocação e na utilização de recursos, considerando:
  - A priorização do benefício máximo de todos os pacientes e do maior número possível de pessoas, reconhecendo que nem todos os pacientes se beneficiam ou necessitarão da utilização de todos os recursos, e, logo, cada recurso deve ser direcionado àqueles que realmente podem se beneficiar de seu uso, segundo as evidências clínicas disponíveis.
  - A identificação e a prioridade daqueles que podem se recuperar utilizando os escassos recursos, antes daqueles que provavelmente vão se recuperar sem a necessidade do uso desses recursos específicos e daqueles cuja recuperação mostra-se improvável, mesmo que submetidos a tratamento intensivo.
  - Os fatores a serem pesados nessa decisão, que incluem:
    i. A avaliação de expectativa de vida a curto e longo prazo (considerando o adoecimento atual e as comorbidades prévias) por meio de instrumentos validados;
    ii. Estimativa de anos de vidas salvos, priorizando pacientes com probabilidade de sobreviver por mais tempo após o tratamento;
    iii. O “direito” de se viver o ciclo completo da vida;
    iv. Os casos identificados como “irreversíveis”, que devem ser alocados para recebimento de Cuidados Paliativos adequados o mais brevemente possível.^( 10 )^
3. Consideração do valor instrumental:
  - Profissionais de saúde são fundamentais no enfrentamento da crise gerada pela pandemia, logo, considerando também os fatores já aqui descritos, considera-se priorizar o atendimento deles, visando à breve recuperação e ao retorno da capacidade de trabalho. O adoecimento da força de trabalho, isto é, dos profissionais de saúde da linha de frente é algo a ser considerado. O conceito que se defende é o de salvar vida de quem pode salvar mais vidas, pois, sem profissionais capacitados, todos os pacientes – e não apenas aqueles com COVID-19 – sofrerão maior risco de mortalidade e terão anos de vida perdidos.
4. Não prioridade por doença: durante uma pandemia, pacientes portadores de doenças outras, que não a doença pandêmica, devem ser triados pelos mesmos critérios de prioridade de atendimento.
5. Oferta de Cuidados Paliativos adequados a todos os que não preencherem critérios de admissão em unidades de terapia intensiva (UTI) devido a quadro grave, com baixa possibilidade de resposta aos tratamentos disponíveis e prognóstico reservado.

O propósito deste documento é fornecer um guia de triagem considerando a possibilidade da demanda de recursos (por exemplo: ventiladores, leitos de UTI) superar a disponibilidade. Sugere-se que estas recomendações devam ser implementadas de imediato, com a anuência da Superintendência/Diretoria de cada hospital, ficando a deflagração operacional a cabo de cada serviço e respeitando-se as particularidades locais. Do mesmo modo, sugere-se que, enquanto durar a situação de excepcionalidade imposta pela pandemia, tal política de alocação de recursos seja mantida, sendo sua suspensão tomada em conjunto com a Superintendência/Diretoria de cada hospital.

### Processo de decisão para alocação de recursos

#### Triagem

A triagem deve ser baseada não na presença da suspeita diagnóstica do COVID-19, mas para todos em condições críticas, para que o acesso seja de maneira igualitária.

O médico que cuida do paciente não deve ser o que decide sobre os critérios de triagem. Recomenda-se que o processo decisório do nível de prioridade seja realizado por pelo menos duas referências administrativas do hospital (por exemplo: supervisor da UTI e chefe do pronto atendimento). Uma vez definido o nível de prioridade, o paciente ou seu representante legal deve ser comunicado quanto ao recurso destinado para o caso. Também é imprescindível que esse processo decisório seja registrado no prontuário do paciente.

#### Critérios de avaliação

Importante ressaltar que o processo de triagem não se baseia em etnia, poder aquisitivo, percepções de qualidade de vida, deficiência intelectual, *status* social, presença de comorbidades específicas ou sexo. A triagem inicial baseia-se em critérios de sobrevida, realizada por meio de escalas, como a de fragilidade clínica de Rockwood et al.,^( 11 )^( Tabela 1 ). Trata-se de uma ferramenta de acesso de fragilidade física, cognitiva e funcional, que reflete tanto comorbidades quanto reserva fisiológica, podendo ser aplicada a todos os indivíduos adultos, independente da idade ou do tipo de doença de base, permitindo que ocorra equidade na avaliação. Fragilidade em pacientes de UTI é fator de risco para mortalidade intra-hospitalar e também de não retorno ao lar.^( 12 , 13 )^A alocação de recursos, então, baseia-se na pontuação da escala escolhida, gerando o grau de prioridade ( Tabela 2 ).

**Tabela 1:** Escala de fragilidade clínica de Rockwood et al.( ^11^ )

Escala de fragilidade	Descrição
1. Em ótima forma	Robusto, ativo, cheio de energia e motivado. Geralmente se exercita regularmente e tem a melhor forma dentro de sua faixa etária
2. Bem	Não tem doença ativa, mas menos em forma que a categoria 1
3. Bem com comorbidades tratadas	Sintomas relacionados à doença de base são bem controlados
4. Aparentemente vulnerável	Apesar de não ser dependente, geralmente se queixa de ser mais lento ou tem sintomas relacionados à comorbidade
5. Fragilidade leve	Tem alguma dependência de outras pessoas para atividades instrumentais (transporte, compras, preparo de refeições, lidar com finanças, usar telefone, gerenciar medicações e tarefa domésticas)
6. Fragilidade moderada	Precisa de ajuda para atividades básicas da vida diária (alimentar, ir ao banheiro, escolher roupa, higiene pessoal, continência esfincteriana, vestir, tomar banho, andar e transferir)
7. Fragilidade intensa	Totalmente dependente de outros para as atividades básicas da vida diária

**Tabela 2:** Alocação de recursos segundo escala de fragilidade clínica de Rockwood et al.( ^11^ )

Grau de prioridade	Rockwood	Alocação
Alta prioridade	1-3	UTI/ semi
Prioridade intermediária	4-6	*ICU trial*
Baixa prioridade	7	Enfermaria – Cuidados Paliativos

UTI: unidade de terapia intensiva; *ICU trial* : tentativa de tratamento em unidade de terapia intensiva.

Se houver empate na pontuação, a decisão pode basear-se no critério de estimativa de anos salvos e ser profissional de saúde envolvido nos cuidados de pacientes com COVID-19. Este último critério não considera que a vida desse profissional é mais valiosa do que a de outras pessoas, mas se dá por seu valor instrumental, como força-tarefa para auxiliar no cuidado de outros indivíduos quando se recuperar. A participação em estudos clínicos relacionados à COVID-19 também pode ser utilizada como critério para alocação de recursos, em um entendimento de recompensa, pela disposição em fazer parte de um estudo que pode trazer benefício aos demais.

## ICU TRIAL

Nos casos em que é optado por uma tentativa de tratamento em UTI ( *ICU trial* ), recomenda-se que fique claro, desde o início da internação em UTI, que existe um tempo em que se é esperado que o paciente melhore, e, caso isso não aconteça, esse dispositivo será retirado por falha terapêutica. O *Sequential Organ Failure Assessment* (SOFA) parece ser um dos fatores prognósticos de mortalidade intra-hospitalar durante a pandemia ( Tabela 3 ).^( 14 , 15 )^Pacientes com SOFA ascendente e, principalmente, com SOFA >10 devem ser candidatos à limitação terapêutica (exemplos: limitação de infusão de DVA, hemodiálise, ventilação mecânica e manobra de reanimação cardiopulmonar). Tendo em vista o comportamento em ventilação mecânica, são propostos, ao menos, 7 dias de terapia para avaliação da retirada de suporte, que deve ser feita por dois profissionais de saúde que não estão no cuidado direto do paciente, a fim de aumentar a objetividade e reduzir o estresse moral da equipe. Recomenda-se acionar a equipe de Cuidados Paliativos e a psicologia nestas situações, além de permitir que familiares participem da fase final de vida presencialmente (limitado a dois acompanhantes) ou por videoconferência.

**Tabela 3:** Sequential Organ Failure Assessment Score

SOFA	0	1	2	3	4
PaO_2_/FiO_2_, mmHg	≥400	<400	<300	<200 (com suporte ventilatório)	<100 (com suporte ventilatório)
Plaquetas, x10^3^/mm^3^	≥150	<150	<100	<50	<20
Bilirrubinas, mg/dL	<1,2	1,2-1,9	2,0-5,9	6,0-11,9	≥12,0
Cardiovascular, drogas em µg/kg/minuto	PAM ≥70mmHg	PAM <70mmHg	Dopamina ≤5 ou dobutamina (qualquer dose)	Dopamina 5-15 ou epinefrina ≤0,1 ou norepinefrina ≤0,1	Dopamina >15 ou epinefrina >0,1 ou norepinefrina >0,1
Escala de coma de Glasgow	15	13-14	10-12	6-9	≤6
Creatinina, mg/dL Débito urinário	<1,2	1,2-1,9	2,0-3,4	3,5-4,9 ou diurese <500mL/d	≥5,0 ou diurese <200mL/d

SOFA: *Sequential Organ failure Assessment* ; PaO_2_/FiO_2_: pressão parcial de oxigênio/fração inspirada de oxigênio; PAM: pressão arterial média.

### Indivíduos de baixa prioridade

Aqueles alocados como baixa prioridade devem receber controle de sintomas e suporte psicossocial em enfermaria. A equipe de Cuidados Paliativos pode auxiliar na coordenação do controle de sintomas, em conjunto com o médico responsável pelo caso, assim como facilitar o processo de comunicação com o paciente e seus familiares.

Em situações de morte iminente, sugere-se a permissão de dois acompanhantes ou videoconferência.

Em situações nas quais o paciente ou seu representante legal solicitem a desospitalização, para ser evitado isolamento social e, mesmo com alto risco de mortalidade, fique em companhia de seus familiares, recomendamos o acionamento da equipe de Cuidados Paliativos. A equipe tem a função de orientar sobre riscos e benefícios desta opção, e esclarecer sobre possíveis estratégias no controle de sintomas e cuidados pós-óbito.
